# Contralateral Effect following Intravitreal Brolucizumab Injection in Diabetic Macular Edema

**DOI:** 10.1155/2022/3755249

**Published:** 2022-01-28

**Authors:** Somnath Chakraborty, Jay Umed Sheth

**Affiliations:** ^1^Retina Institute of Bengal, Siliguri, India; ^2^Surya Eye Institute and Research Center, Mumbai, India

## Abstract

The authors describe a novel case of a 48-year-old male with bilateral diabetic macular edema (DME) who underwent intravitreal injection (IVI) of brolucizumab in the left eye. At four weeks, the patient demonstrated a bilateral response by way of improvement in the best-corrected visual acuity (BCVA) and reduction in the central macular thickness (CMT) in both eyes. Further studies on the ocular and systemic assays of the brolucizumab molecule are warranted to evaluate its systemic escape and to better understand the pharmacokinetics behind the bilateral effect.

## 1. Introduction

Antivascular endothelial growth factor (anti-VEGF) therapy has become the treatment of choice for retinal vascular disorders such as diabetic macular edema (DME) [1, 2]. Pegaptanib sodium (Macugen®, Eyetech/OSI Pharmaceuticals, New York, NY, USA), ranibizumab (Lucentis®; Genentech, S. San Francisco, CA/Roche, Basel, Switzerland), aflibercept (Eylea®, Regeneron, Tarrytown, NY), and brolucizumab (Beovu®; Novartis, Basel, Switzerland) are four antivascular endothelial growth factor (anti-VEGF) agents that the US Food and Drug Administration (FDA) has approved for intraocular usage [3–5]. Amongst them, brolucizumab is the latest to receive approval for neovascular age-related macular disorders (nAMD). In the case of DME, two phase 3 clinical studies, KESTREL and KITE, are underway to assess the role of brolucizumab, while its off-label usage in eyes with recalcitrant DME has already been described [6, 7].

The contralateral effect of intravitreal injections, including ranibizumab, bevacizumab, aflibercept, triamcinolone acetonide, and dexamethasone implant, has been described [8–12]. In our case report, we demonstrate the bilateral response following unilateral intravitreal injection (IVI) of brolucizumab in a patient with DME, which remains unreported in the literature.

## 2. Case Report

**Figure 1:**
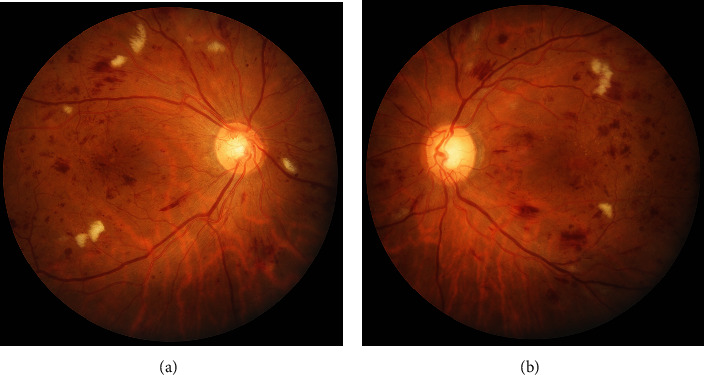
Fundus photographs of both the eyes demonstrating severe nonproliferative diabetic retinopathy (NPDR) with clinically significant macular edema (CSME) (a, b).

**Figure 2:**
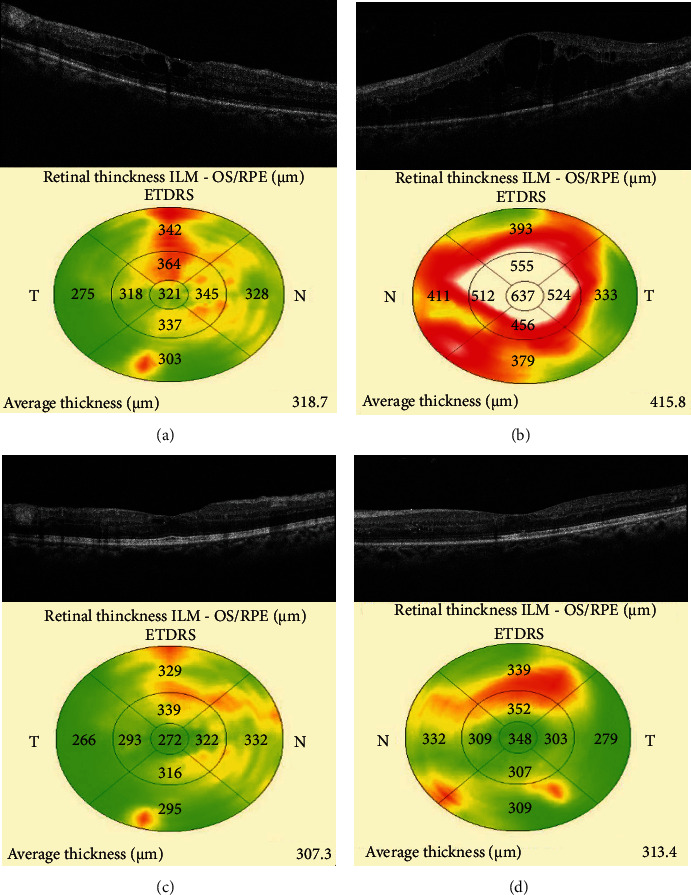
Spectral-domain optical coherence tomography scans of both the eyes (OU) showing the presence of cystoid macular edema (CME) at baseline (a, b). One month after intravitreal brolucizumab therapy, the patient demonstrated a bilateral reduction in the CME (c, d).

A 48-year-old male patient with non-insulin-dependent diabetes mellitus (NIDDM) for 10 years presented with diminution of vision in both eyes (OU) for three months. His best-corrected visual acuity (BCVA) was 20/60 in the right eye (OD) and 20/120 in the left eye (OS). OU anterior segment was normal. Based on fundus examination, he was diagnosed with OU severe nonproliferative diabetic retinopathy (NPDR) with clinically significant macular edema (CSME) involving the OS more than the OD (Figures 1(a) and 1(b)). The presence of CSME was confirmed on the spectral-domain optical coherence tomography (SD-OCT) with a central subfield thickness (CST) of 321 *μ*m in OD and 637 *μ*m in OS (Figures 2(a) and 2(b)). At baseline, his HbA1c levels were 6.8% with normal renal parameters (blood urea: 20 mg/dL, serum creatinine: 0.9 mg/dL). For economic constraints, the patient underwent IVI brolucizumab only in the OD, while OS was observed. At one month, the patient had bilateral improvement in the visual acuity (OU: 20/40) with a reduction in the CSME, although only the OS was injected. On SD-OCT, the CST reduced to 272 *μ*m in the OD and 248 *μ*m in the OS, i.e., a quantitative decrease of 15.26% and 61.07% in the OD and the OS, respectively (Figures 2(c) and 2(d)). There were no ocular or systemic adverse events after the brolucizumab therapy.

## 3. Discussion

Diabetic macular edema is a leading cause of vision impairment on a global scale [13]. The extensive use of intravitreal anti-VEGF therapy has transformed DME care. Newer molecules such as aflibercept and brolucizumab have a longer half-life and durability, thus having the capability of reducing the overall treatment burden [7]. Prospective phase 3 studies (KITE and KESTREL) are being conducted to investigate IVI brolucizumab's role in the management of DME, based on the encouraging results of phase 3 trials testing it in the treatment of nAMD [6, 7]. Brolucizumab was found to be noninferior to aflibercept in terms of mean change in visual acuity at one year in the interim results of the KITE and KESTREL studies, which were reported at the end of 2020 [7]. Chakraborty et al. have demonstrated excellent anatomical and visual improvement with brolucizumab in eyes with recalcitrant DME [7]. Based on these encouraging results, our patient was offered treatment with IVI brolucizumab.

To the best of our knowledge, no reports of intravitreal brolucizumab affecting the contralateral eye have been published. Furthermore, the exact mechanism by which it may occur has yet to be determined. Other anti-VEGF medications have occasionally been shown to produce similar contralateral effects [8–12]. The most universally recognized hypothesis is the systemic escape of the molecule which can then lead to a contralateral effect [9]. Microvascular permeability and molecule size have been shown to be inversely related in studies [14]. For this reason, the brolucizumab molecule, which has the lowest weight amongst all anti-VEGF agents (brolucizumab (26 kDa) versus bevacizumab (149 kDa) versus ranibizumab (48 kDa) versus aflibercept (110 kDa)), can easily enter the systemic circulation and have a contralateral effect. Additionally, diabetic retinopathy is associated with altered inner blood-retinal barrier and increased vascular permeability [2]. These dysfunctional retinal vascular changes may also influence the systemic absorption of intravitreally administered medications.

One drawback of our case is that we did not get drug quantification assays from the aqueous, vitreous, and serum samples. Moreover, improvements in SD-OCT parameters and BCVA in the contralateral eye may be due to the disease's natural progression. However, in our case, the patient's systemic parameters, including the glycemic status and the renal profile, were well controlled at baseline. As a result, a reduction in the quantum of macular edema in the contralateral eye of up to 16% is extremely unlikely due to systemic parameter management. Thus, this contralateral effect in all probability is secondary to a systemic crossover of the brolucizumab molecule. While this systemic crossover proved beneficial in our situation, it is crucial to remember that it can also be associated with harmful systemic side effects. This demands extensive investigation into the pharmacokinetics of the brolucizumab molecule, which guards against unanticipated ocular and systemic adverse effects.

In conclusion, our case report highlights the bilateral response after IVI brolucizumab therapy in a single eye most probably due to the systemic escape phenomenon. More research on the ocular and systemic assays of the brolucizumab molecule is needed to assess its systemic escape and better understand the pharmacokinetics of the bilateral action.

## Data Availability

No data were used to support this study.
